# Antiviral potential of zinc ion: mechanisms and applications in viral infections

**DOI:** 10.3389/fmicb.2026.1770051

**Published:** 2026-05-08

**Authors:** Junxiang Zhao, Jincheng Li, Jiayi Wang, Leiliang Zhang

**Affiliations:** 1 Department of Clinical Laboratory Medicine, The First Affiliated Hospital of Shandong First Medical University & Shandong Provincial Qianfoshan Hospital, Jinan, Shandong, China; 2 Department of Pathogen Biology, School of Clinical and Basic Medical Sciences, Shandong First Medical University & Shandong Academy of Medical Sciences, Jinan, Shandong, China

**Keywords:** antiviral, host-virus interaction, mechanisms of action, viral protein, zinc ion

## Abstract

Zinc ion (Zn^2+^) is essential bioactive constituent in food and plays a multifaceted role in modulating antiviral activities against various viruses including retroviruses, picornaviruses, herpesviruses, coronaviruses, and hepatitis viruses. This review synthesizes existing literature highlighting the diverse antiviral properties of Zn^2+^ and its viable application as an adjunct rather than monotherapeutic agent in viral infections. We outline three principal mechanisms through which Zn^2+^ exerts its antiviral effects: direct interaction with viral proteins, interference with viral genetic replication processes, and modulation of host cellular environments to hinder viral replication. We further elaborate on the physiological role of Zn^2+^ in human nutrition, its dietary intake pathways, and the influence of nutritional zinc status on the antiviral efficacy of Zn^2+^. This establishes a crucial connection between dietary zinc homeostasis and the host's antiviral defense. Future research directions should focus on elucidating the complex interplay between Zn^2+^ and viral dynamics, assessing zinc's geographical and age-related distribution in relation to viral infections, and developing clinical strategies to enhance Zn^2+^ efficacy against a broader range of viruses. This review is essential for advancing our understanding of zinc's role in food chemistry and its critical contributions to human health and antiviral defense mechanisms.

## 1 Introduction

Zinc is a vital trace element in human nutrition and the second most abundant trace metal in the human body after iron, with total body content ranging from 2 to 4 grams and plasma concentrations between 12 to 16 μM (Wessels et al., 2021). It is essential for human growth, the proper functioning of the immune system, and the maintenance of cellular homeostasis, with dietary intake being the sole source of endogenous zinc supply for humans (Frederickson et al., 2005). The recommended dietary allowance of zinc for adult males is 11 mg/day and 8 mg/day for adult females, with increased requirements (11–12 mg/day) for pregnant and lactating women, and 3–11 mg/day for children based on age (Wessels et al., 2021). Dietary zinc is predominantly absorbed in the small intestine via zinc transporters (Kambe et al., 2014; Maret, 2017), with rich dietary sources including oysters, red meat, poultry, whole grains, and legumes. However, phytates in plant-based foods can chelate zinc and reduce its bioavailability by up to 60% (Rink and Gabriel, 2000). Zinc deficiency is a global public health issue, particularly prevalent in low-income regions, elderly populations, children, and individuals with malabsorption syndrome (Rink and Gabriel, 2000; Wessels et al., 2021).

Additionally, research has demonstrated that zinc plays a crucial role in mediating the anti-inflammatory and antioxidant effects of interferon (IFN). In recent years, as studies on zinc nanomaterials have intensified (Gökmen et al., 2024), the significance of zinc ion (Zn^2+^) has often been overshadowed. However, Zn^2+^ can influence various viral infections by impacting these critical processes. Moreover, the presence of Zn^2+^ in food highlights their importance not only for nutrition but also for immunity and overall health, as dietary zinc status directly modulates intracellular zinc bioavailability and thus the antiviral potential of Zn^2+^–zinc-deficient individuals exhibit blunted antiviral responses to exogenous zinc supplementation, while adequate dietary zinc enhances intracellular zinc accumulation and viral inhibition (Chasapis et al., 2021; Wessels et al., 2021). Notably, Zn^2+^ has not been validated as an effective single therapeutic agent for any viral infection in clinical settings; its antiviral effects are most pronounced when used as an adjunct therapy in combination with antiviral drugs, ionophores, or nutritional intervention (Derwand and Scholz, 2020; Fantini et al., 2020). This review aims to summarize the specific mechanisms through which Zn^2+^ affect different types of viruses, systematically address the contradictions and limitations of zinc as a monotherapy, and clarify the correlation between dietary zinc nutrition and Zn^2+^ antiviral efficacy, providing a theoretical foundation for future research on the effects of Zn^2+^ on viral pathogens and advancing our understanding of their antiviral potential within the context of food systems.

## 2 Retrovirus

Retroviruses, such as human immunodeficiency virus (HIV), exhibit a spherical morphology with a diameter ranging from 100 to 120 nm. The viral structure is encapsulated in a lipoprotein envelope, while the nucleocapsid core has a bullet-like shape. This core contains two identical positive-stranded RNA genomes, which are surrounded by the nucleocapsid protein p7 (NCp7) and the capsid protein p24. Additionally, HIV carries essential enzymes such as reverse transcriptase (RT), integrase, and protease. HIV is transmitted predominantly through three routes: sexual contact, blood exposure, and vertical transmission from mother to child. The virus leads to the suppression of the immune system in host cells, which can ultimately result in mortality due to immune failure.

**Figure 1:**
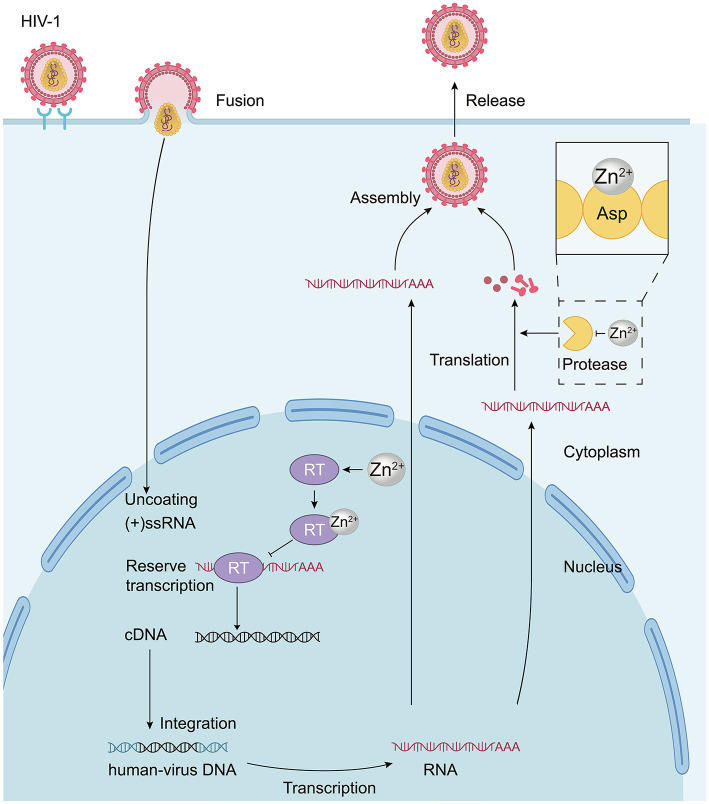
The mechanism by which Zn^2+^ inhibits HIV-1. During the replication stage of the viral genetic material, Zn^2+^ exhibits a high affinity for reverse transcriptase (HIV-RT). The binding of Zn^2+^ to reverse transcriptase forms a hybrid complex (RT-Zn^2+^) that restricts RT activity for an extended period, significantly reducing the catalytic extension rate and resulting in a so-called “dead-end complex.” This complex inhibits the reverse transcription process of the virus. During the translation stage, Zn^2+^ can bind to the zinc-binding site at the aspartic acid residues of HIV protease, interfering with the production of viral proteins. Thus, Zn^2+^ affects viral replication through these two mechanisms, yielding antiviral effects.

Research has shown that Zn^2+^ possesses a unique inhibitory effect on retroviruses, including HIV. However, no clinical evidence supports the use of zinc as a single therapy for HIV infection. Monotherapy with zinc salts fails to reduce viral load or delay disease progression in HIV-positive patients, and high-dose zinc supplementation may even interfere with the absorption of antiretroviral drugs (ARVs) such as tenofovir and emtricitabine (Pannecouque et al., 2010). Zn^2+^ can disrupt the RT activity of these viruses (Fenstermacher and DeStefano, 2011) and displace Zn^2+^ from the zinc finger domains present within retroviruses, thereby reducing viral replication (Tummino et al., 1996; Berthoux et al., 1999; Pannecouque et al., 2010; Figure 1). This effect has been observed in both HIV (particularly subtype 1) and equine infectious anemia virus (EIAV). In HIV, RNA selection and packaging during assembly involve two highly conserved “CCHC” zinc finger domains of the nucleocapsid proteins, where Zn^2+^ is coordinated to maintain stability (Bombarda et al., 2007). Various inhibitors targeting NCp7, such as N,N′-bis(4-ethoxycarbonyl-1,2,3-thiadiazol-5-yl)-benzene-1,2-diamine (NV038) (Pannecouque et al., 2010) and disulfide benzamide (DIBA-1) specifically interfere with these zinc finger structures (Tummino et al., 1996; Berthoux et al., 1999). These compounds can expel Zn^2+^ from NCp7's zinc finger, leading to conformational changes that result in NCp7 inactivation. This process inhibits the maturation of viral proteins and consequently hinders viral replication during transcription (De Rocquigny et al., 1997), ultimately resulting in the formation of non-infectious viral particles with antiviral properties. However, such zinc chelators alone show limited *in vivo* efficacy, requiring combination with ARVs to achieve synergistic viral inhibition (Pannecouque et al., 2010).

The RT of HIV (HIV-RT), like other RTs, exhibits both nucleotide polymerase and RNase H activities (Levin et al., 2005). Both activities require divalent cations as cofactors. Zn^2+^ not only facilitates the polymerase and RNase H activities but also effectively obstructs HIV-RT catalysis. By forming a stable RT-Zn^2+^-(primer-template) hybrid complex with high affinity, Zn^2+^ can limit the action of RT, significantly reducing the catalytic extension rate. Thus, Zn^2+^ inhibits HIV-RT synthesis indirectly by creating a stable dead-end complex, where the kinetics of incorporation are rendered extremely slow, thereby hampering viral replication and manifesting antiviral effects (Fenstermacher and DeStefano, 2011). Additionally, Zn^2+^ can interact with a zinc-binding site at the aspartic acid residues of HIV protease, ensuring stable coordination that inhibits HIV-1 protease at neutral pH (York et al., 1993; Figure 1). This interaction disrupts viral protein maturation and replication, leading to further antiviral effects. Overall, zinc primarily interferes with the transcription processes of retroviruses like HIV.

In addition, recent advances in high-resolution imaging and structural biology have revised the classical model of HIV-1 replication (Müller et al., 2021). The intact or near-intact HIV-1 capsid can traverse the nuclear pore complex into the nucleus, where reverse transcription occurs within the nuclear compartment, and uncoating takes place in the nucleus near the integration site (Shen et al., 2021; Müller et al., 2022). The identification of such intranuclear replication sites has profound implications for understanding the antiviral mechanism of Zn^2+^. Zn^2+^ must cross the nuclear envelope to exert its inhibitory effects on these critical intranuclear steps, making intranuclear antiviral activity critically dependent on the nuclear membrane permeability and intranuclear targeting efficiency of Zn^2+^.

Zn^2+^ inhibits retroviruses through conserved molecular mechanisms in both the host cytoplasm and nucleus. In both subcellular compartments, Zn^2+^ acts primarily through zinc ion displacement and modulation of metal ion-dependent viral enzymatic activities, thereby suppressing viral zinc-finger domains and divalent cation-dependent viral enzymes. For instance, Zn^2+^ can displace the intrinsic Zn^2+^ within the zinc-finger motif of NCp7 in both the cytoplasm and nucleus, inducing conformational inactivation. It can also bind to RT to form a dead-end complex, significantly slowing polymerization kinetics and effectively blocking viral genome replication (Fenstermacher and DeStefano, 2011). Ultimately, Zn^2+^ converges on core antiviral outcomes by inhibiting viral genome replication and protein maturation (Bombarda et al., 2007; Pannecouque et al., 2010).

Nonetheless, pronounced differences exist between cytoplasmic and nuclear Zn^2+^ regarding target types, inhibitory efficiency, and regulatory susceptibility. In the cytoplasm, Zn^2+^ targets mainly incoming free viral particles, incompletely translocated viral cores, and free viral enzymes in the cytosol, acting during the early stages of viral replication. In contrast, the primary targets within the nucleus are the nuclear-translocated viral reverse transcription complexes and nuclear-imported HIV-1 integrase, which forms functional complexes with LEDGF/p75 and associates with host chromatin, corresponding to the mid-to-late stages of viral replication (Maertens et al., 2003).

Regarding inhibitory efficiency, Zn^2+^ in the cytoplasm directly interacts with incoming viruses, blocking infection at an early stage. The basal concentration of free cytoplasmic Zn^2+^ is relatively low, in the picomolar to nanomolar range, and exogenous supplementation can more readily elevate Zn^2+^ to effective inhibitory concentrations (Colvin et al., 2010). In contrast, the nuclear envelope barrier impedes Zn^2+^ entry, which occurs primarily via specific transporters or passive diffusion. Free nuclear Zn^2+^ is derived from the cytoplasm and maintained at levels comparable to or lower than those in the cytoplasm (Beyersmann, 2002).

Distinct regulatory interference further distinguishes the two compartments. The antiviral activity of intranuclear Zn^2+^ is susceptible to modulation by host nuclear zinc-binding proteins and chromatin-modifying factors, which rapidly sequester free Zn^2+^ and reduce its bioavailability, thereby maintaining nuclear zinc homeostasis (Beyersmann, 2002). In contrast, the cytoplasmic zinc regulatory network exerts weaker interference against exogenously supplemented Zn^2+^, allowing Zn^2+^ to act more stably on viral targets (Colvin et al., 2010).

## 3 Picornavirus

The *Picornaviridae* family encompasses a group of non-enveloped viruses with particle diameters ranging from 20 to 30 nm. These picornaviruses contain single-stranded, positive-sense RNA that directs the synthesis of a polyprotein precursor. Subsequently, this precursor undergoes cleavage by the virus's own proteases, 3C and 2A, resulting in the formation of L, P1, P2, and P3 products. Further cleavage of P1, P2, and P3 yields four structural proteins (VP1, VP2, VP3, and VP4) and seven non-structural proteins (2A, 2B, 2C, 3A, 3B, 3C, and 3D). Notable examples of picornaviruses include coxsackie virus (CV), human rhinovirus (HRV), enterovirus (EV), poliovirus, and Mengo virus, among others. Infections caused by these viruses can lead to a range of infectious and potentially fatal diseases. For instance, coxsackie virus B3 (CVB3) is particularly associated with viral myocarditis and can contribute to dilated cardiomyopathy, heart failure, and aseptic meningitis (Beck et al., 1990), significantly impacting children. HRV is known to cause respiratory illnesses and can trigger asthma and chronic obstructive pulmonary disease, posing heightened risks to children and the elderly, who are populations with high rates of dietary zinc deficiency (Rink and Gabriel, 2000).

Research has demonstrated that Zn^2+^ exhibits inhibitory effects on these viruses; however, its monotherapeutic efficacy is highly variable and depends on concentration, resulting in contradictory outcomes in clinical and preclinical studies. High-dose zinc not only fails to enhance antiviral activity but also impairs picornavirus polyprotein processing through unknown mechanisms, which ultimately leads to reduced viral inhibition (Krenn et al., 2009). Therefore, focusing on CVB3 and HRV as representative cases, scientists explored the mechanisms by which Zn^2+^ inhibits picornaviruses during multi-protein processing. It was observed that Zn^2+^ facilitated the action of pyrrolidine dithiocarbamate (PDTC), impacting proteasome activity, which in turn influenced multi-protein processing and exerted antiviral effects. For CVB3, Zn^2+^ promoted PDTC to chelate metal ions and enhance their cellular entry (Krenn et al., 2005; Lanke et al., 2007). Zn^2+^ acted as an inhibitor of E3 ubiquitin ligase (Hayakawa et al., 2003) and might directly inhibit proteasome activity or the ubiquitin-proteasome-dependent activities of molecules such as D1, p53, p21, and MKP-1(Kim et al., 2004; Si et al., 2005), thereby inhibiting viral replication. Notably, CVB3 infection upregulates ZnT5 expression to reduce intracellular zinc levels, which counteracts the antiviral effects of exogenous zinc monotherapy. This necessitates the use of ionophores in combination to overcome viral-mediated zinc sequestration (Frisk et al., 2007).

**Figure 2:**
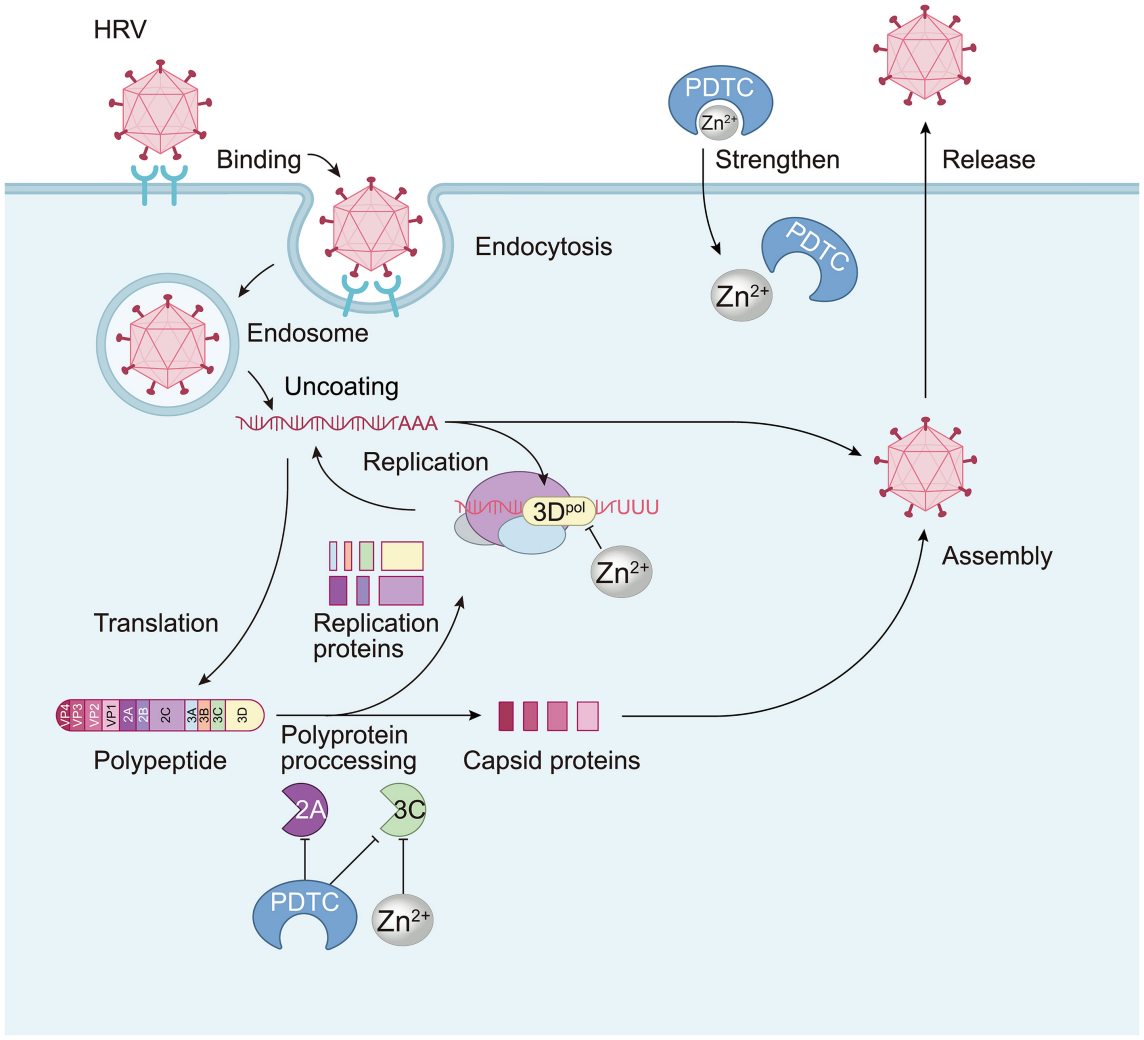
The anti-HRV mechanism of Zn^2+^. Zn^2+^ primarily exerts its effects by mediating and enhancing the impact of PDTC on proteasome activity, which influences the processing of multiple proteins. PDTC can impair the processing of the P1 protein by inhibiting viral proteases 2A and 3C, affecting subsequent cleavage and viral protein expression. Additionally, Zn^2+^ can also affect 3C protease without significantly influencing 2A. For HRV16, Zn^2+^ inhibits the activity of its RNA-dependent RNA polymerase (3D^pol^) *in vitro*, thereby suppressing polymerase function and interfering with RNA replication.

Similarly, for HRV, prior findings have indicated that Zn^2+^ can inhibit viral replication (Korant et al., 1974), with several mechanisms identified (Figure 2). Through PDTC, Zn^2+^ obstructs the processing and expression of viral multi-proteins, with PDTC inhibiting the processing of the P1 protein by impairing the activity of viral proteases 2A and 3C, thus affecting further cleavage and subsequent viral protein expression. Additionally, PDTC was found to reduce the expression of 2A, impacting the functioning of the cellular translation initiation factor eIF4GI and the cleavage of cytokeratin 8 (Gaudernak et al., 2002). Concurrently, Zn^2+^ inhibits the activity of 3C protease (Cordingley et al., 1989), thereby blocking the post-translational cleavage of precursor proteins (Korant et al., 1974; Korant and Butterworth, 1976), although it does not exert a direct influence on protease 2A.

Moreover, Zn^2+^ and PDTC also modulate viral RNA synthesis by affecting multi-protein processing: PDTC has been shown to attenuate the synthesis of both positive and negative strands of viral RNA. Additionally, it has been documented that Zn^2+^ can inhibit the activity of HRV16 RNA-dependent RNA polymerase (RdRp) 3D polymerase (3D^pol^), thereby obstructing polymerase function and viral replication *in vitro* (Hung et al., 2002). Compounds like PT and HK, which share similarities with PDTC, also function as zinc carriers and perform analogous roles, with zinc uptake being reversible. However, excessive zinc concentrations can impair the processing of picornaviruses' multi-proteins, although the precise mechanisms remain inadequately defined. On one hand, viral protease function may be directly hindered; on the other, Zn^2+^ could induce folding issues in viral multi-proteins, resulting in precursors that cannot be properly separated (Krenn et al., 2009). For enterovirus EV-D68, Zn^2+^ appears to function in a somewhat different antiviral capacity, with findings suggesting that the PDTC zinc carrier can encourage zinc influx into cells to bolster antiviral capabilities (Liu et al., 2021). Although specifics remain unexamined, the underlying mechanisms likely align with those affecting picornaviruses. Zn^2+^ further inhibits EV-D68 by interrupting its entry and release pathways (Liu et al., 2021). Notably, no clinical trials have shown that zinc monotherapy is effective for EV-D68 or CVB3 infections, and nutritional zinc supplementation is recommended only as a preventive measure for high-risk children (Rink and Gabriel, 2000; Liu et al., 2021).

## 4 Herpes simplex virus (HSV)

HSV displays distinctive morphological features typical of herpesviruses, with mature virus particles measuring approximately 180 nm in diameter. Its core structure contains a double-stranded DNA genome approximately 150 kb long, encoding over 80 proteins. HSV-1 is commonly transmitted through contact, predominantly affecting the lips, and poses a particular threat to newborns; potential consequences include blisters, ulcers, and systemic infections that can lead to severe inflammatory responses or mortality. Additionally, there is evidence suggesting a link between HSV-1 and Alzheimer's disease (Itzhaki et al., 1997). In contrast, HSV-2 spreads through direct contact with skin and mucosal surfaces or sexually, causing genital herpes and associated complications. Furthermore, it is recognized as a risk factor for the spread and progression of HIV-1 (Nagot et al., 2007). Importantly, HSV-2 can infect newborns during delivery, leading to severe local damage and encephalitis affecting the skin, eyes, and mouth, potentially resulting in sepsis and death.

**Figure 3:**
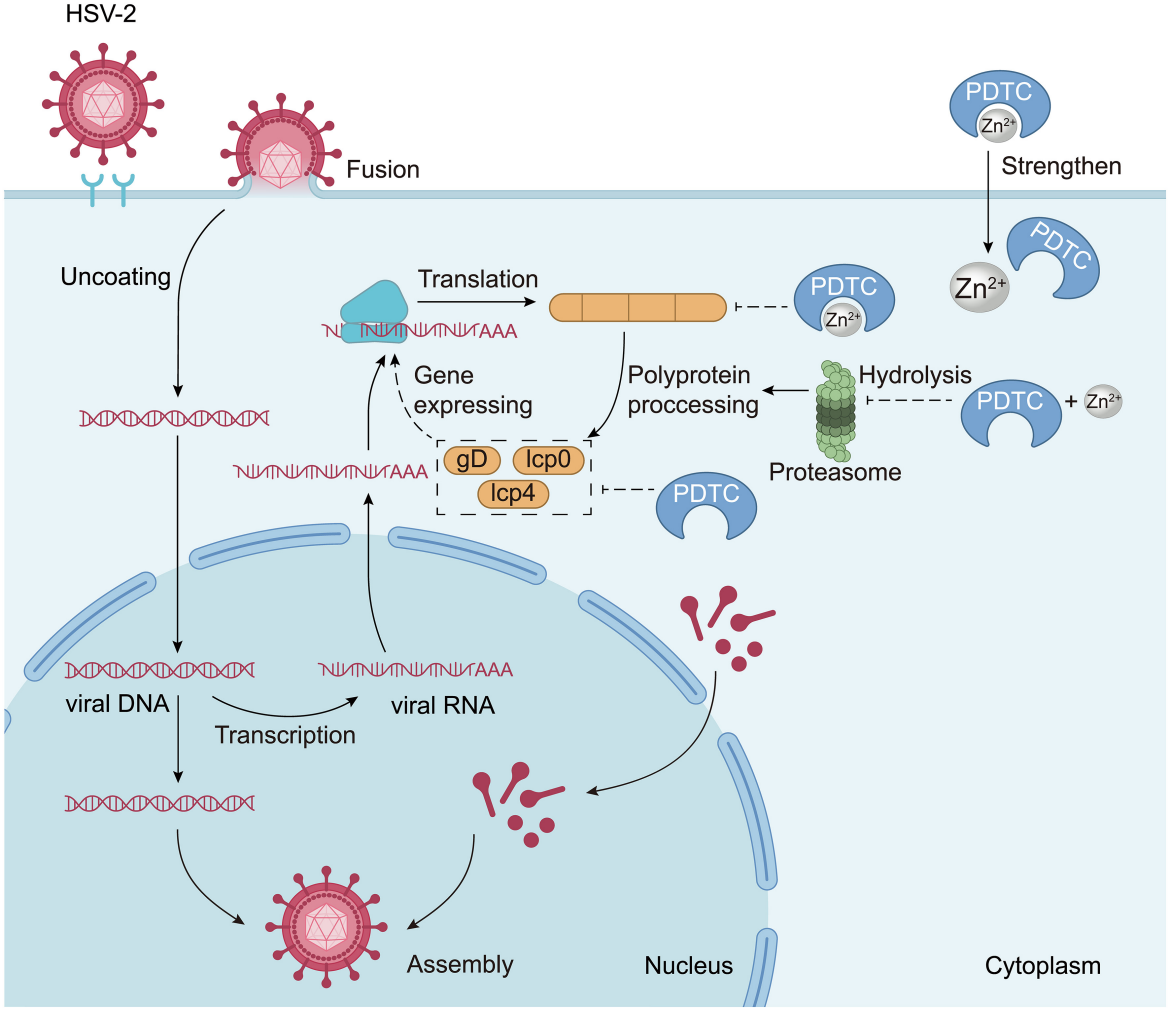
The anti-HSV-2 mechanism of Zn^2+^. Similarly, Zn^2+^ primarily acts by mediating and enhancing PDTC activity. Specifically, Zn^2+^ can inhibit the expression of the HSV membrane protein gD, leading to decreased gD expression and reduced viral replication. Zn^2+^ also inhibits ICP0 and ICP4, which are critical for viral replication. Moreover, PDTC can chelate Zn^2+^, facilitating their transport into mammalian cells. Both PDTC and Zn^2+^ can mediate the dysregulation of the intracellular ubiquitin-proteasome system (UPS) and inhibit proteasome-dependent protein hydrolysis, including the inhibition of the 26S proteasome.

Zn^2+^ and PDTC exhibit inhibitory effects on HSV-1 and HSV-2 (Kümel et al., 1990; Arens and Travis, 2000), but the clinical efficacy of zinc monotherapy for HSV infections is context-dependent and remains a topic of debate. Clinical trials have shown that topical zinc salts, such as zinc sulfate gel or zinc oxide/glycine cream, can significantly reduce the duration of cold sore lesions caused by HSV-1 when applied early (Godfrey et al., 2001). However, oral zinc supplementation shows no significant effect on HSV-2 genital herpes in preclinical models, indicating that the route of administration is critical for efficacy. Research has demonstrated that zinc salts can effectively inactivate HSV *in vitro* (Arens and Travis, 2000), but this effect may be influenced *in vivo* by factors such as skin or mucosal penetration and tissue binding. The antiviral mechanisms of Zn^2+^ against HSV mirror those observed for picornaviruses. Initial findings suggest that Zn^2+^ can mediate and amplify PDTC activity (Kim et al., 1999) and inhibit the expression of the HSV membrane protein gD, which is pivotal for gene expression during the intermediate and late stages of the viral lifecycle (Figure 3). A reduction in gD expression correlates with a decline in viral replication. Additionally, the immediate early regulatory proteins ICP0 and ICP4 play crucial roles in HSV replication (Everett, 1984; O'Hare and Hayward, 1985), with PDTC impacting immediate early gene expression and viral replication through inhibition of ICP4 expression and modulation of ICP0 localization during the early stages of the viral lifecycle (Qiu et al., 2013; Figure 3).

PDTC can also disrupt the ubiquitin-proteasome system in host cells, hindering the proteasome-dependent degradation of IκB-α and thereby inhibiting HSV-facilitated NF-κB activation, which is critical for viral replication (Schreck et al., 1992; Ziegler-Heitbrock et al., 1993). Furthermore, by chelating Zn^2+^, PDTC enhances the transport of zinc into mammalian cells (Kim et al., 2004; Lanke et al., 2007) while inhibiting 26S proteasome cleavage activities (Krenn et al., 2005), subsequently blocking the ubiquitin-coupled protein loss and IκB-α degradation induced by HSV-2, ultimately diminishing gD expression and HSV-2 replication (Qiu et al., 2013). PDTC's promotion of Zn^2+^ influx leads us to hypothesize that Zn^2+^ also disrupts the ubiquitin-proteasome balance and proteasome-dependent proteolysis (Qiu et al., 2013).

Overall, Zn^2+^, alongside PDTC as an exemplary carrier, exhibit antiviral properties against both picornaviruses and herpes simplex viruses through specific mechanisms. Primarily, Zn^2+^ mediates PDTC's antiviral effects, and due to zinc's reversible nature, utilizing zinc chelators like EDTA diminishes PDTC's antiviral efficacy, underscoring zinc's crucial role in this activity. Additionally, Zn^2+^ can infiltrate cells via the carrier for enhanced effects, with the predominant mode of action revolving around interference with multi-protein processing. However, zinc monotherapy is not recommended for HSV infections due to its poor *in vivo* efficacy, and a combination with antiviral drugs, such as acyclovir, is necessary to achieve synergistic inhibition. Furthermore, regarding drug efficacy and resistance, a combination of zinc sulfate and heparin appears to be superior to zinc sulfate alone, as heparin retains its activity even after 20 passages, whereas acyclovir produces a completely resistant virus after only a single passage. The use of the genotoxic DNA polymerase inhibitor acyclovir should be restricted to severe cases of HSV (Kümel et al., 1995). It is also important to note that distinct differences in the actions of Zn^2+^ related to HAV within picornaviruses will be discussed further.

## 5 Coronavirus

Coronaviruses are enveloped, positive-sense RNA viruses characterized by a spherical shape, measuring 80-140 nm in diameter. They can be transmitted through respiratory droplets and various other means, leading to serious respiratory illnesses such as severe pneumonia and acute respiratory distress syndrome, particularly affecting the elderly and children, who are populations with high rates of zinc deficiency and impaired zinc absorption (Wessels et al., 2021). In light of the global outbreak of severe acute respiratory syndrome coronavirus 2 (SARS-CoV-2), recent years have seen increased investigation into the effects of Zn^2+^ on coronaviruses. Extensive clinical evidence demonstrates that zinc monotherapy is ineffective for COVID-19, while zinc supplementation in combination with ionophores (e.g., hydroxychloroquine) or antiviral drugs (e.g., nirmatrelvir) shows synergistic efficacy in reducing disease severity (Derwand and Scholz, 2020; Fantini et al., 2020; Imran et al., 2022; Tao et al., 2022).

Specifically, Zn^2+^ can reversibly inhibit the synthesis and elongation activity of the coronavirus RdRp, a key enzyme within the viral replication-transcription complex (RTC). This inhibition reduces the RNA synthetic activity of the RTC and subsequently impedes viral replication (te Velthuis et al., 2010). Zn^2+^ may mediate the effects of compounds such as PDTC or PT to exert antiviral effects, potentially functioning similarly to their roles in other RNA viruses previously discussed, although this area requires further study.

Furthermore, zinc's effects on the RNA synthesis of the SARS-CoV RTC extend beyond the initiation stage and may also disrupt the elongation phase, potentially by directly impairing the interaction between SARS-CoV RdRp and its RNA template (te Velthuis et al., 2010). Additionally, studies indicate that the inhibitory actions of Zn^2+^ may apply to a broader range of positive-sense RNA viruses in the Nidovirales order, including SARS-CoV, other human coronaviruses, equine arteritis virus (EAV), and porcine reproductive and respiratory syndrome virus (PRRSV). Zn^2+^ inhibits the replication of these viruses through mechanisms similar to those observed in SARS-CoV. For non-SARS-CoV coronaviruses, however, zinc primarily influences the RNA initiation phase without markedly affecting elongation (te Velthuis et al., 2010). Notably, zinc deficiency is associated with increased severity and mortality of COVID-19, as individuals with low zinc levels exhibit reduced ACE2 receptor regulation and impaired interferon production. This underscores the importance of dietary zinc nutrition in defending against coronaviruses, rather than relying solely on zinc monotherapy (Imran et al., 2022).

**Figure 4:**
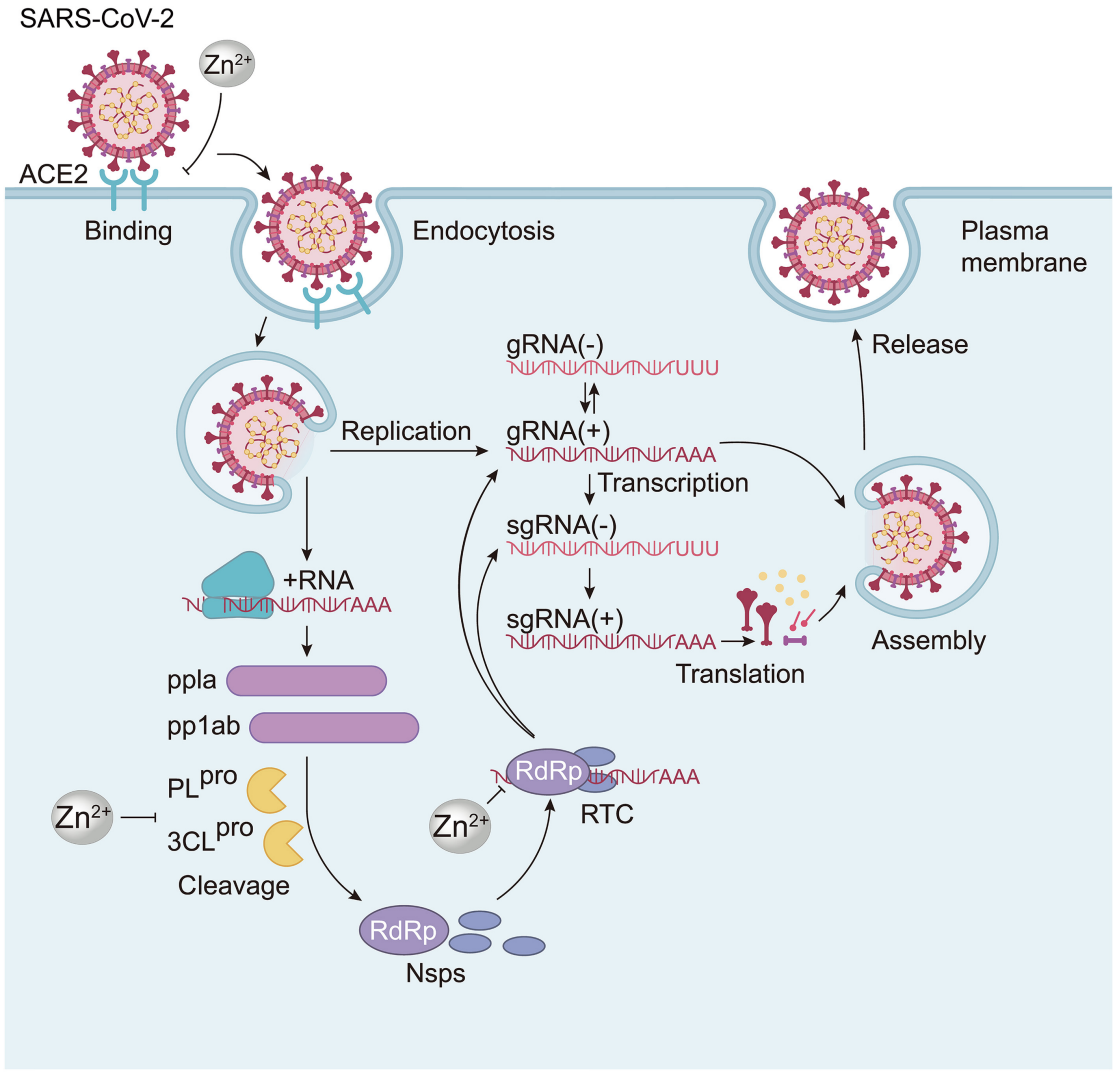
The mechanism by which Zn^2+^ inhibits SARS-CoV-2. Zn^2+^ can prevent the virus from entering cells by reducing the expression of ACE2 receptors. Additionally, Zn^2+^ inhibits the activity of two important proteases, PL^pro^ and 3CL^pro^, which are involved in processing and translating the virus's proteins. Concerning genetic material replication, Zn^2+^ reversibly inhibits the synthesis activity of coronavirus RdRp, the core enzyme of the viral multiprotein replication and transcription complex (RTC). This inhibition affects RNA synthesis activity in the RTC and subsequent elongation processes, ultimately suppressing viral replication.

In the specific case of SARS-CoV-2, Zn^2+^ can downregulate ACE2 receptor expression by inhibiting sirtuin-1 (SIRT-1), thereby reducing the viral entry into host cells (Imran et al., 2022). Additionally, chloroquine (CQ) and hydroxychloroquine (HCQ) can function as zinc carriers, facilitating zinc transport and enhancing its antiviral effects by disrupting the pH-dependent stages of SARS-CoV-2 replication, such as fusion and uncoating (Derwand and Scholz, 2020; Fantini et al., 2020). This combination proves to be significantly more effective than zinc monotherapy, which does not reduce ACE2 expression or viral entry *in vivo* (Imran et al., 2022). Moreover, Zn^2+^ inhibits the activity of PL^pro^ and 3CL^pro^, two crucial proteases encoded by the virus that are responsible for processing and translating multiple viral proteins, further contributing to the inhibition of SARS-CoV-2 replication (Tao et al., 2022; Figure 4). A recent study has demonstrated that zinc gluconate combined with hinokitiol (a zinc ionophore) inhibits SARS-CoV-2 replication *in vitro* and in animal models, while zinc gluconate alone shows no significant effect (Tao et al., 2022). This confirms the necessity of combination therapy to enhance the antiviral efficacy of zinc against coronaviruses.

## 6 Flavivirus

Dengue virus (DENV), which belongs to the *Flavivirus* genus, is a single-stranded positive-sense RNA virus approximately 11 kb in size. It encodes three structural proteins and seven non-structural proteins. DENV is primarily transmitted by *Aedes aegypti* and *Aedes albopictus* mosquitoes and is mainly found in tropical and subtropical regions. Infection can cause systemic pain, severe hemorrhagic symptoms, and can potentially lead to shock or death.

The crystal structure of the DENV NS5 protein has revealed a zinc-binding site within its RdRp domain, suggesting that zinc plays a structural role in polymerase activity, which the virus exploits for replication (Yap et al., 2007). Consequently, zinc may be critical for the polymerase function of dengue virus. Zinc chelators, such as N,N,N′,N′-tetra (2-pyridylmethyl)ethylenediamine (TPEN), have been used to simulate zinc deficiency, inducing reactive oxygen species (ROS) and inhibiting dengue replication. This demonstrates a specific inhibition of DENV infection, an effect that can be reversed with zinc supplementation (Khan et al., 2021). Research indicates that zinc chelation may trigger endoplasmic reticulum stress responses and activate NF-κB and its downstream targets, leading to the inhibition of viral RNA synthesis and effectively blocking dengue virus replication without affecting other RNA viruses (Kar et al., 2019; Khan et al., 2021). Notably, when Zn^2+^ is supplemented, the inhibitory effect of TPEN on DENV infection is negated, which is unfavorable for viral inhibition (Khan et al., 2021).

Hepatitis C virus (HCV) particles exhibit a spherical morphology, with an average diameter of 55-65 nm, featuring a nucleocapsid and a lipid envelope adorned with surface spikes. The virus's single-stranded positive-sense RNA genome is approximately 9.6 kb long and encodes a polyprotein divided into structural proteins (core, E1, E2) and non-structural proteins (p7, NS2, NS3, NS4A, NS4B, NS5A, NS5B) (47). Humans are the sole hosts, and transmission occurs primarily through blood, potentially leading to hepatitis C, which has high incidence and mortality rates, along with the risk of liver cirrhosis or cancer (Zoulim et al., 2003).

Various studies suggest that Zn^2+^ can inhibit the replication of HCV RNA and have been reported to inhibit the activity of HCV NS5B RdRp, in a dose-dependent manner (Yuasa et al., 2006). However, HCV encodes a self-protease, NS2-3, which cleaves the ns2/ns3 junction and plays a crucial role in the virus's lifecycle. Zn^2+^ can activate the enzyme activity (Thibeault et al., 2001), and the addition of Zn^2+^ enhances the activity when combined with the NS3 protease, facilitating the cleavage of non-structural proteins. The protease activities of NS2/3 and NS3 depend on the same Zn^2+^ bound to NS3, with Zn^2+^ serving a catalytic role in the NS2/3 protease and a structural role in the NS3 protease (Vega et al., 2013). Although Zn^2+^ may influence HCV protein processing, it still exhibits an overall antiviral effect (Yuasa et al., 2006; Kumar et al., 2023). Based on existing literature, it is speculated that the inactivation effect of Zn^2+^ on RdRp may be insufficient, and other mechanisms, including the enhancement of IFN-α to boost cellular immune responses and the antioxidant properties of Zn^2+^, likely play a significant role (Grüngreiff and Reinhold, 2010).

Notably, dietary zinc deficiency is linked to persistent HCV infection and a poor treatment response. Up to 48.4% of patients with chronic hepatitis C demonstrate hypozincemia, which correlates with impaired antiviral immunity and a reduced response to interferon-based therapies (Gupta et al., 2019). Nutritional zinc supplementation as an adjunct to antiviral therapy has been shown to improve outcomes. However, the doses used in clinical studies often exceed the recommended dietary allowance of 15 mg/day, emphasizing that zinc is utilized as a therapeutic adjunct rather than merely a nutritional correction. These findings highlight the importance of targeted zinc supplementation in the management of HCV, rather than advocating for zinc monotherapy (Yuasa et al., 2006; Kumar et al., 2023).

## 7 Alphavirus

Alphaviruses are members of the *Togaviridae* family and are characterized as positive-sense single-stranded RNA viruses with an envelope. Each virus possesses a capsid membrane and has a diameter of approximately 70 nm. Its genome encodes four non-structural proteins (nsP1-4) as well as structural proteins, including E1 (Strauss and Strauss, 1994). The chikungunya virus (CHIKV) can cause chikungunya fever and associated arthritis (De Souza et al., 2025), while the eastern and western equine encephalitis viruses may lead to human encephalitis (Reyna and Weaver, 2023), primarily transmitted by mosquitoes. Zinc acetate has been shown to inhibit CHIKV *in vitro* by targeting the CHIKV nsP2 protease (Saha et al., 2018), which affects the processing of non-structural peptide precursors necessary for viral replication. Literature indicates that zinc salts may influence several steps following infection, including viral entry, proteolytic processing of various proteins, replication, and assembly of viral particles. The inhibitory effect of zinc salts on negative-strand RNA synthesis is particularly pronounced, suggesting that zinc may impact replication or pre-replication stages; however, the specific mechanisms remain unclear and warrant further investigation (Davuluri et al., 2024). Furthermore, Zn^2+^ demonstrates a high affinity for the E1 domain of the virus, binding to this region to inhibit pH-dependent liposome fusion of the E1 domain in the alphavirus envelope glycoproteins. This action potentially impedes infections from viruses such as CHIKV, Sindbis virus, and Semliki forest virus (Bracha and Schlesinger, 1976; Corver et al., 1997; Davuluri et al., 2024). Notably, zinc salts combined with magnesium salts demonstrate synergistic antiviral effects against CHIKV *in vitro*, whereas zinc monotherapy shows limited efficacy. This supports the need for combination strategies in the treatment of alphavirus infections (Davuluri et al., 2024).

## 8 Hepatitis A virus (HAV)

HAV is the only species within the *Hepatovirus* genus. It can lead to viral hepatitis A but does not cause chronic hepatitis. The primary hosts for HAV are humans and other vertebrates, with a particular tendency to infect children and adolescents, populations that have increased zinc requirements and a high risk of deficiency (Rink and Gabriel, 2000). The virus is typically excreted in the feces of infected individuals at the end of the incubation and acute phases, and it primarily spreads via the fecal-oral route through contaminated food, water, and seafood (Martin and Lemon, 2006). Research has shown that zinc chloride can inhibit the expression of mitogen-activated protein kinase 3 (MAP2K3), which subsequently downregulates HAV replication in human hepatocytes, thereby exhibiting an antiviral effect (Kanda et al., 2021). Additionally, zinc chloride significantly upregulates mitogen-activated protein kinase 12 (MAPK12) while downregulating several associated genes, including baculovirus IAP repeat 3 (BIRC3), interleukin-1 β (IL1B), proline-serine-threonine phosphatase interacting protein 1 (PSTPIP1), prostaglandin-endoperoxidase 2 (PTGS2), PYD and CARD domain-containing protein (PYCARD), and tumor necrosis factor (TNF). This collective modulation may enhance the anti-HAV activity of interferon (Kanda et al., 2020).

Notably, zinc chloride inhibits HAV replication in human hepatoma cells at the dosage of 5 μM that are achievable through adequate dietary zinc intake (Kanda et al., 2021). The antiviral effects observed *in vitro* are mediated by the suppression of MAP2K3 expression and modulation of inflammatory gene expression, suggesting a mechanistic basis for the role of nutritional zinc homeostasis in HAV defense. However, it has not been directly evaluated whether these *in vitro* effects translate into clinical benefits or whether high-dose zinc supplementation provides any additional advantages, as all current evidence is derived from cell culture studies rather than clinical trials (Kanda et al., 2021).

## 9 Hepatitis E virus (HEV)

HEV is a single-stranded, positive-sense RNA virus measuring approximately 7.2 kb in length. It features a 5′ terminal unstructured region and a 3′ terminal structural region. The virus particles are spherical, lack a capsid, and have a diameter of about 27–38 nm, characterized by fibrils and notches on their surfaces. HEV infection can lead to hepatitis E, primarily affecting young and middle-aged adults. The incidence is lower among children and the elderly, while pregnant women are particularly susceptible to the disease (Kumar et al., 2014), representing a group with increased zinc requirements and a heightened risk of deficiency (Wessels et al., 2021). Although hepatitis E rarely progresses to chronic hepatitis, it carries a high mortality risk. Currently, there is no clinical evidence supporting zinc monotherapy for HEV infection. Observational studies in pregnant women, a population at high risk for severe HEV outcomes, have found that lower circulating zinc concentrations during early pregnancy are associated with an increased risk of HEV infection, suggesting that zinc deficiency may predispose individuals to the virus (Kmush et al., 2016; Nair et al., 2016; Kaushik et al., 2017). These findings highlight a potential role for adequate zinc nutrition in HEV prevention; however, direct evidence showing that zinc supplementation reduces disease severity in pregnant women with HEV is lacking and requires further clinical investigation. HEV is primarily transmitted through the fecal-oral route. Research has shown that zinc salts, including zinc sulfate and zinc acetate, can inhibit HEV replication. Initial studies indicated that these zinc salts specifically inhibited the replication of genotype 1 and genotype 3 HEV replicons, as well as genotype 1 HEV infectious virus, in a dose-dependent manner. Further investigations using various mutants in Huh7 cells revealed that zinc salts affect the newly identified viral protein ORF4 and directly inhibit the activity of the viral RdRp, resulting in reduced viral replication (Nair et al., 2016; Kaushik et al., 2017). Therefore, zinc ion should only be regarded as a potential adjunct therapy for HEV, rather than a standalone treatment.

## 10 Respiratory syncytial virus (RSV)

**Figure 5:**
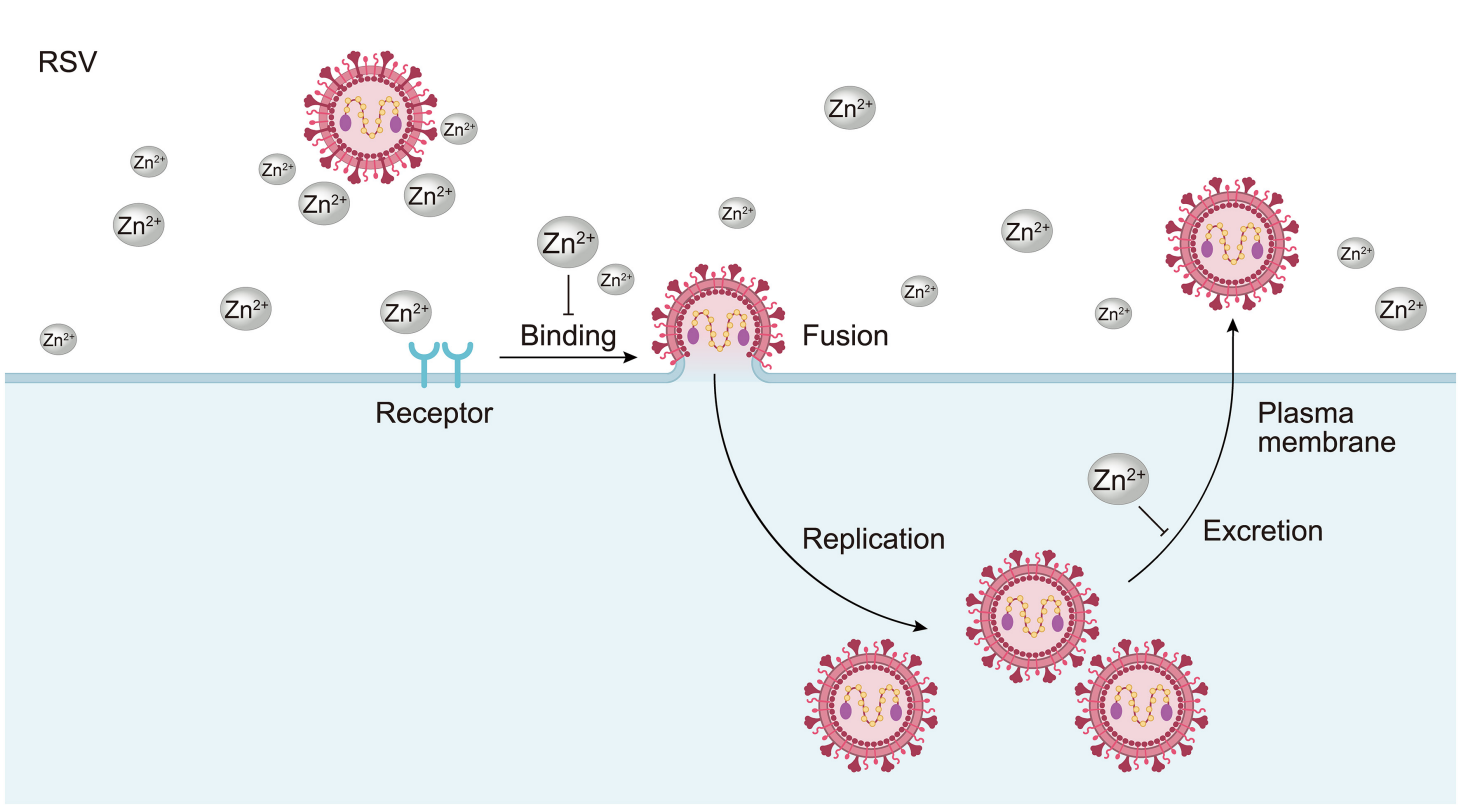
The anti-RSV mechanism of Zn^2+^. Zn^2+^ does not exert antiviral effects by inhibiting viral replication directly; instead, it alters the cell's ability to support viral replication. Zn^2+^ or zinc salts deposit on or within cell monolayers, preventing virus adsorption and subsequent plaque formation. At the same time, they contribute to virus penetration and elimination, inhibiting viral replication and transmission into cells.

RSV belongs to the *Pneumoviridae* family. It is a spherical, enveloped virus with a diameter of approximately 150 nm. The viral genome consists of a non-segmented linear, negative-sense single-stranded RNA of about 15.2 kb in length. RSV primarily affects children, the elderly (especially those over 65), and individuals with compromised immune systems, potentially leading to acute upper respiratory tract infections. In severe cases, it can result in lower respiratory tract infections, such as bronchitis and pneumonia. Existing literature suggests that Zn^2+^, including zinc sulfate and zinc acetate, has been investigated in relation to RSV. Previous studies indicate that Zn^2+^ is rarely absorbed by cells (Arens and Travis, 2000). Instead, it is believed that these ions may alter cellular conditions in a way that supports viral replication *in vitro* rather than directly inhibiting it. Zn^2+^ can inhibit viral adsorption; for instance, Zn^2+^ or salts that settle on or within the cell monolayer can prevent virus adsorption and subsequent plaque formation (Figure 5). Additionally, Zn^2+^ can influence viral penetration and egress, thereby inhibiting viral replication and transmission. This inhibitory effect is concentration-dependent and exhibits specificity (Suara and Crowe, 2004; Figure 5). Notably, topical zinc (e.g., nasal sprays) demonstrates mild efficacy in reducing the duration of RSV symptoms in children; however, oral zinc monotherapy has no significant impact on viral load or disease severity (Suara and Crowe, 2004), underscoring the limited utility of zinc as a standalone treatment for RSV.

## 11 Conclusions and prospects

Through our investigation and synthesis of existing literature, we have identified Zn^2+^ as a broad-spectrum antiviral agent, demonstrating significant effects against a diverse array of viruses, including retroviruses, picornaviruses, HSV, coronaviruses, various viruses within the Nidovirales order, as well as flaviviruses, RSV, and the well-studied hepatitis viruses HAV and HEV. A critical aspect of this conclusion is the recognition that Zn^2+^ is not an effective standalone therapeutic agent for any viral infection as its antiviral efficacy is limited by poor cellular penetration, concentration-dependent toxicity, viral-mediated zinc sequestration, and contradictory effects on viral replication (e.g., DENV, HCV). Consequently, Zn^2+^, along with commonly used salts such as zinc sulfate and zinc acetate, may serve as valuable adjunct therapies during viral infections, showcasing diverse antiviral properties.

In summarizing the mechanisms of action, we categorize the antiviral effects of Zn^2+^ into three main groups (Table 1). The first group encompasses the direct interaction of Zn^2+^ with critical viral proteins. In this mechanism, Zn^2+^ often operates synergistically with carriers such as PDTC, facilitating cellular transport and enhancing intracellular zinc concentrations, which in turn amplifies antiviral activity. Moreover, Zn^2+^ can potentiate the intrinsic antiviral capabilities of these carriers. For instance, by targeting viral proteases, Zn^2+^ disrupts viral protein function and inhibits protein synthesis, a notable mechanism in picornaviruses and HSV. The second category pertains to the interference with viral genetic information, which is further divided into two subtypes. The first subtype focuses on retroviruses such as HIV, wherein Zn^2+^ interacts with RT, significantly delaying the reverse transcription process and thus inhibiting viral replication. The second subtype includes other RNA viruses, in which Zn^2+^ targets RdRp, impairing its function and preventing replication. Notably, Zn^2+^ exhibits distinct strategies for inhibiting RNA replication in specific viruses, as detailed in previous sections. The third category involves reducing the ability of host cells to support viral replication, wherein Zn^2+^ interferes with viral adsorption, penetration, and dissemination rather than directly targeting viral components. A pertinent example is RSV, where Zn^2+^ creates a barrier on the host cell surface that obstructs viral entry and exit. Conversely, for HIV, the presence of zinc finger structures allows zinc chelators to induce zinc ejection, disrupting viral replication. While zinc finger structures are evident in various viruses, research has predominantly focused on their structural roles, leaving their interactions with Zn^2+^ less explored.

**Table 1:** Antiviral mechanisms of zinc ions targeting viral proteins and host factors.

Group	Target	Virus	Detailed mechanism	References
Direct interaction of Zn^2+^ with critical viral proteins	NCp7's zinc finger	HIV	Displace Zn^2+^ → conformational inactivation → Inhibit assembly and maturation	Pannecouque et al. (2010)
	E3 ubiquitin ligase; viral proteases 2A and 3C; membrane protein gD and 26S proteasome cleavage; PLpro, 3CL^pro^; nsP2 protease	CVB3, HRV, HSV, SARS-CoV-2, CHIKV	Synergize with zinc ionophores such as PDTC → inhibit enzymatic activity → block polyprotein processing	Gaudernak et al. (2002); Kim et al. (2004); Krenn et al. (2005); Saha et al. (2018); Tao et al. (2022)
	Reverse transcriptase	HIV	Create a stable dead-end complex → block reverse transcription	Fenstermacher and DeStefano (2011)
Interference with viral genetic information	Envelope glycoprotein (gD/E1)	HSV, Alphavirus	Inhibit membrane fusion/adsorption → block invasion	Corver et al. (1997); Qiu et al. (2013)
	Ubiquitin-proteasome system	CVB3, HSV	Inhibit IκBα degradation → downregulate NF-κB → weaken replication signaling	Si et al. (2005); Qiu et al. (2013)
	Gene expression including MAP2K3 and MAPK12	HAV	Downregulate multiple gene expressions → enhance the anti-HAV activity of interferon	Kanda et al. (2020)
Reducing the ability of host cells to support viral replication	Cellular receptor (ACE2)	SARS-CoV-2	Inhibit sirtuin-1 to downregulate ACE2 receptor expression → reduce viral cell entry	Imran et al. (2022)
	Cell surface barrier	RSV, EV-D68	Deposit on or inside cell monolayers → blockadsorption/penetration/release → inhibit spread	Suara and Crowe (2004)

When examining research outcomes related to the antiviral effects of Zn^2+^ on picornaviruses and HSV, several successful interventions emerge alongside notable failures. A consistent theme across successful studies is the role of Zn^2+^ in interfering with viral proteases, thereby disrupting the processing of viral polyproteins necessary for replication. However, significant discrepancies arise in the use of Zn^2+^ concentrations. Moderate levels show antiviral activity, while excessive concentrations can harm protein processing. This can undermine the antiviral efficacy. For instance, effective modulation of CVB3 and HRV replication has been documented (Korant et al., 1974; Beck et al., 1990; Suara and Crowe, 2004), yet instances demonstrating high zinc concentrations causing processing failures reveal a critical bottleneck (Krenn et al., 2009). Additionally, the successful disabling of gD protein expression in HSV-2 highlights the effectiveness of Zn^2+^ in disrupting enveloped virus mechanisms, contrasting with challenges faced in enterovirus applications, where variability in effectiveness was noted based on the virus strain and cellular context (Kümel et al., 1990).

Research has also depicted a swift correlation between proper PDTC engagement and enhanced Zn^2+^ transport, leading to promising antiviral effects and revealing a pathway to optimize carrier systems (Krenn et al., 2005; Lanke et al., 2007). Successful outcomes, such as those observed with PDTC modulation and proteasome disruption, reinforce the validity of targeting host cellular pathways alongside direct viral inhibition (Schreck et al., 1992; Ziegler-Heitbrock et al., 1993; Qiu et al., 2013). Conversely, failures often stem from an inadequate understanding of the intricate interplay between Zn^2+^, viral cycles, and potential feedback mechanisms that may exacerbate viral replication under certain conditions. These complexities prompt future inquiries into establishing optimal treatment regimens that precisely balance these variables to maximize antiviral potential while avoiding pitfalls associated with Zn^2+^ therapies. In summary, future studies should aim to refine our understanding of these mechanisms, leveraging successes in the application of Zn^2+^ while learning from setbacks encountered, particularly with HAV's exceptions and the variability observed in other viral contexts. This endeavor will pave the way for targeted antiviral strategies with potential therapeutic applications against a broader spectrum of viral pathogens.

It is also crucial to recognize that Zn^2+^ does not always exert antiviral effects; in certain scenarios, it may facilitate viral replication. For example, Zn^2+^ has been reported to activate viral enzymes such as RdRp in DENV, thereby promoting replication, while zinc chelation can significantly inhibit viral replication. Beyond direct antiviral actions, studies have indicated that Zn^2+^ also mediates indirect antiviral effects through two primary mechanisms: enhancing IFN-α activity to bolster host immunity and acting as antioxidants against viral infections. Due to their indirect and relatively nonspecific nature, these mechanisms were not extensively addressed in this paper.

Therefore, the antiviral efficacy of Zn^2+^ is intricately linked to the participation and regulation of the immune system, forming a bidirectional synergistic antiviral network (Read et al., 2019). Zn^2+^ enhances the host's ability to clear viruses by regulating the development and activation of immune cells, cytokine secretion, interferon signaling pathways, and antioxidant pathways. At the same time, the activation state of the immune system can influence the intracellular distribution and utilization efficiency of Zn^2+^ by regulating the expression of zinc transporters, while viral infections can disrupt this balance and compromise host defense mechanisms.

First, Zn^2+^ is an essential factor for the development and activation of immune cells. Its deficiency leads to a reduction in the number and functional impairment of T cells, B cells, and natural killer (NK) cells (Yao et al., 2025). Studies have confirmed that Zn^2+^ can promote the differentiation of T helper cell 1 (Th1) subsets, enhance the secretion of pro-inflammatory cytokines such as IFN-γ and IL-2, and maintain the balance of antiviral immunity. Regarding NK cells, Zn^2+^ can improve their cytotoxicity and enhance their ability to recognize and eliminate virus-infected cells (Prasad, 2007). A recent clinical study in 2025 found that vegetarians and vegans exhibited downregulated expression of interferon regulatory factor 3 (IRF3) in peripheral blood mononuclear cells due to zinc deficiency, significantly reducing the IFN-α antiviral response. However, short-term oral zinc supplements effectively upregulated IRF3 expression and restored the antiviral secretion capacity of IFN-α, confirming the direct regulatory effect of Zn^2+^ on the innate immune interferon pathway (Vallboehmer et al., 2025).

Second, Zn^2+^ serves as a key regulatory factor in the IFN signaling pathway, amplifying the expression of antiviral genes (such as MX1 and OAS1) by enhancing the production of IFN-α/β and the phosphorylation of its downstream STAT proteins (Read et al., 2019). For example, in HCV infection, Zn^2+^ deficiency leads to a decreased response rate to IFN therapy, while Zn^2+^ supplementation can improve the antiviral therapeutic effect by strengthening the IFN signaling pathway (Ishikawa, 2012). In COVID-19 patients, zinc deficiency is closely associated with insufficient IFN production and a cytokine storm, and zinc supplementation can mitigate lung inflammatory damage by regulating immune balance (Jin et al., 2024).

These indirect mechanisms are closely linked to dietary zinc status. Zinc deficiency impairs IFN production and antioxidant defense, while adequate dietary zinc enhances these pathways, making nutritional zinc supplementation a critical preventive strategy against viral infections (Wessels et al., 2021). Additionally, zinc nutritional supplementation can serve as a preventive measure for populations at high risk of zinc deficiency, such as vegetarians, the elderly, and pregnant women, thereby reducing the risk of viral infection by maintaining normal immune function and Zn^2+^ bioavailability (Yao et al., 2025). Due to their indirect and relatively non-specific nature, these mechanisms were not extensively addressed in this paper.

Notably, the relationship between Zn^2+^ and viral dynamics is complex and bi-directional. Zn^2+^ is an essential trace element that must be obtained through dietary sources (Frederickson et al., 2005). However, the majority of zinc within cells exists in bound forms, necessitating entry through specific proteins located in the plasma membrane (Yamasaki et al., 2007). The primary families of proteins that facilitate zinc entry are the ZIP and zinc transporter (ZnT) families. ZIP proteins primarily transport Zn^2+^ into the cytoplasm, while ZnT proteins are responsible for exporting zinc out of the cytoplasm or into organelles (Kambe et al., 2014; Maret, 2017). Dietary zinc bioavailability directly influences the expression and activity of these transporters: zinc deficiency upregulates ZIP transporters to enhance absorption, while zinc excess upregulates ZnT transporters to facilitate excretion. An examination of viruses interacting with Zn^2+^ reveals that certain viruses can influence zinc transporters. For instance, CVB3 upregulates the expression of the zinc transporter ZnT5 upon host cell infection, reducing cytoplasmic zinc levels and counteracting the antiviral effects of Zn^2+^ (Frisk et al., 2007). Similarly, SARS-CoV-2 can modulate zinc transporters to lower intracellular zinc levels, thereby facilitating viral infection (Chasapis et al., 2021). In DENV, the zinc transporter ZIP8 shows increased activity following host cell infection, promoting zinc influx that supports viral replication (Panwar et al., 2021). These viral-mediated alterations in zinc homeostasis further limit the efficacy of zinc monotherapy, as exogenous zinc cannot surmount viral-induced zinc sequestration without the aid of ionophores or transporter modulators.

Looking ahead, future research should focus on investigating the correlation between dietary zinc intake and Zn^2+^ levels in various populations, as well as the geographical and age-related distribution of virus-induced diseases. This would facilitate an assessment of whether these observations align with the established understanding of zinc-virus interactions discussed herein. Geographically, most viruses included in this discussion have a global presence and do not adhere to specific epidemic regions, complicating efforts to establish a direct link between zinc levels and viral distribution. Additionally, zinc deficiency is concentrated in low-income regions characterized by plant-based diets, which overlap with high incidence rates of viral infections, suggesting a potential causal link that warrants further epidemiological investigation.

In terms of age demographics, infants, adolescents, and the elderly require higher zinc intake and often experience zinc deficiency. Adults, especially pregnant and lactating women, also have increased zinc needs, making them more susceptible to deficiency (Rink and Gabriel, 2000). Evidence indicates that picornaviruses such as HRV and CVB3 predominantly infect children and the elderly. Coronaviruses and RSV primarily target these age groups as well. HAV) predominantly infects children, while HEV and HSV-2 are more common among pregnant women. These trends suggest a strong correlation between Zn^2+^ nutritional status and susceptibility to viral infections across various age groups. Therefore, future research should focus on nutritional zinc supplementation as a preventive strategy in these high-risk populations, rather than on zinc monotherapy as a therapeutic approach.

Moving forward, it is imperative to delve deeper into the mechanisms through which Zn^2+^ exerts antiviral effects against a broader spectrum of viruses, emphasizing their clinical applications and therapeutic potentials in viral treatments. It is therefore essential to consider the role of zinc ions in combination with ionophores, antiviral agents, and nutritional interventions, rather than as monotherapies. Additionally, future investigations should focus on how viruses may influence Zn^2+^ distribution within host cells, guiding experimental designs to confirm these interactions and potentially uncover new therapeutic strategies. Given the essential role of zinc in human health and its dietary sources, this exploration could enhance our understanding of zinc's contribution to food chemistry and its critical role in combating viral infections.
